# Supplementary material for: The Theoretical and Experimental Investigation of the Fluorinated Palladium β-Diketonate Derivatives: Structure and Physicochemical Properties
Source: Molecules. 2022 Mar 28;27(7):2207. doi: 10.3390/molecules27072207 (PMC9000314; doi:10.3390/molecules27072207)
Supplement: Supplementary file 1 [file molecules-27-02207-s001.zip › molecules-1647498-supplementary.pdf]

molecules

MDPI

Supplementary File

The Theoretical and Experimental Investigation of the Fluorinated Palladium β-Diketonate Derivatives: Structure and Physicochemical Properties

Svetlana I. Dorovskikh [1,2,*], Denis E. Tryakhov [2], Darya D. Klyamer [1], Alexander S. Sukhikh [1,2], Irina V. Mirzaeva [1,2], Natalia B. Morozova [1] and Tamara V. Basova [1,]

[1] Nikolaev Institute of Inorganic Chemistry, Siberian Branch of Russian Academy of Sciences, 3 Acad. Lavrentiev Ave., 630090, Novosibirsk, Russia; dorov@niic.nsc.ru (S.I.D.); klyamer@niic.nsc.ru (D.D.K.); a_sukhikh@niic.nsc.ru (A.S.S.); dairdre@gmail.com (I.V.M.); mor@niic.nsc.ru (N.B.M.); basova@niic.nsc.ru (T.V.B.)

[2] Novosibirsk State University, 2 Pirogova Str., 630090, Novosibirsk, Russia;tryakhovd@gmail.com (D.E.T.)

* Correspondence: dorov@niic.nsc.ru (S.I.D.); Tel.: +7-383-3309556 (S.I.D.)

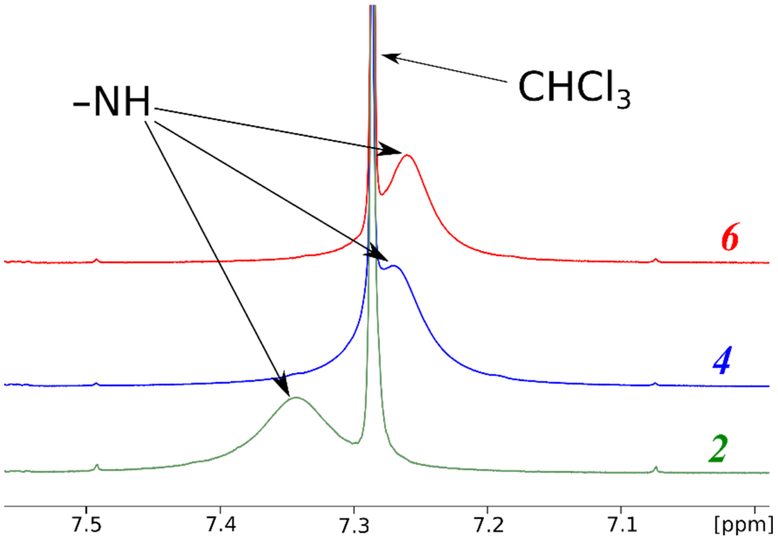

Figure S1. ^{1}H NMR signal of -NH group.

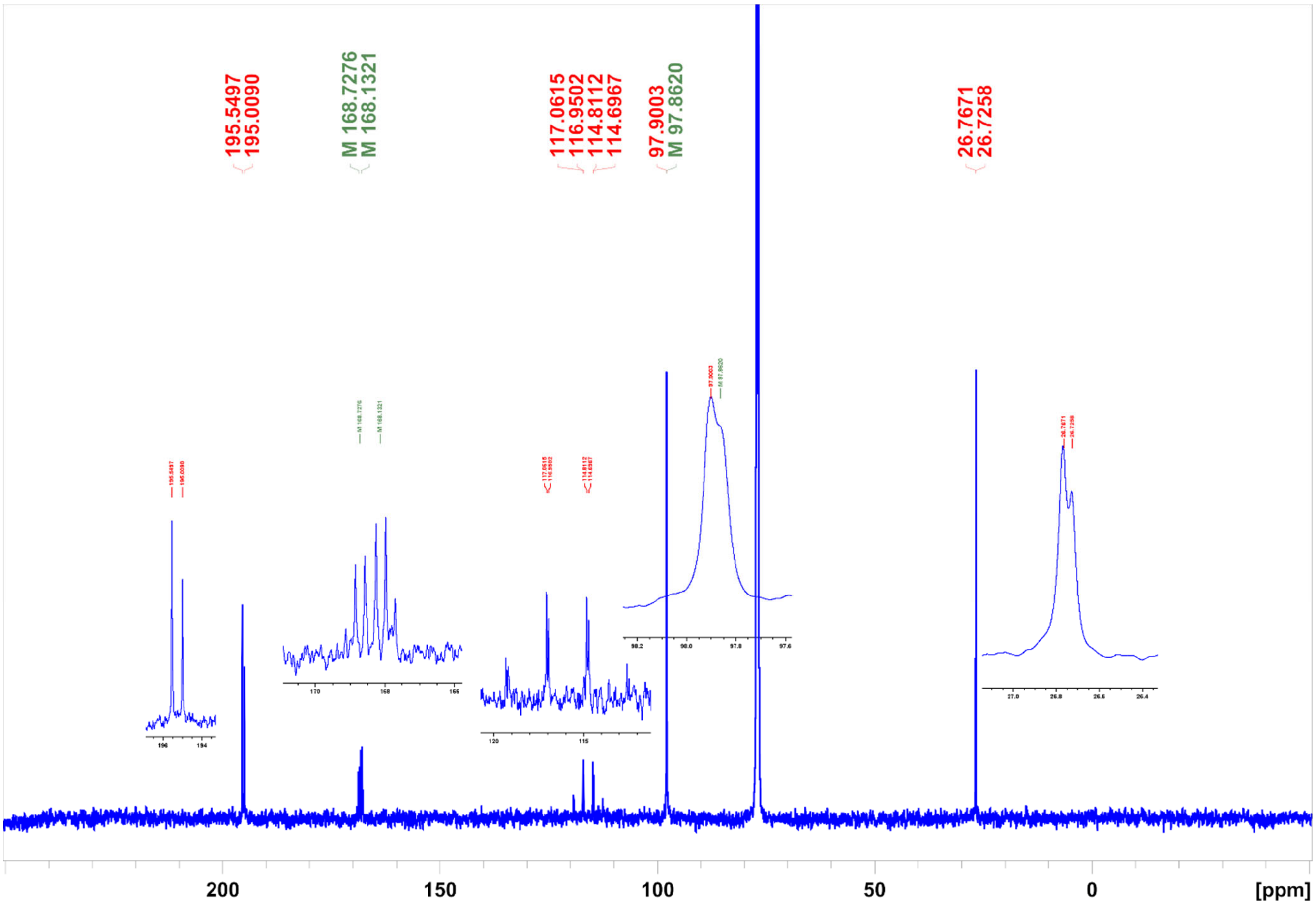

Figure S2. ^{13}C NMR spectrum of -NH group in 1.

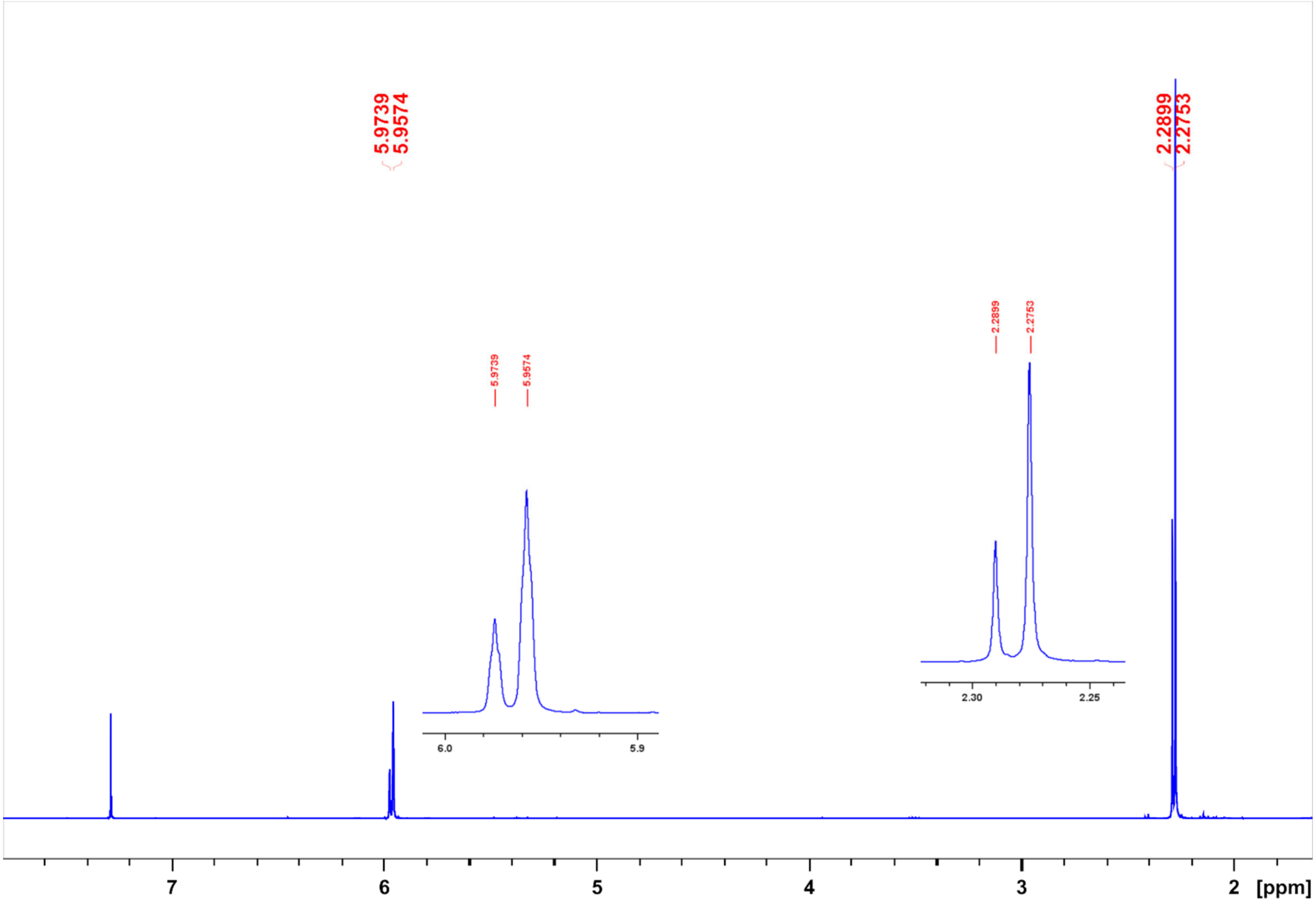

Figure S3. ^{1}H NMR spectrum of 3.

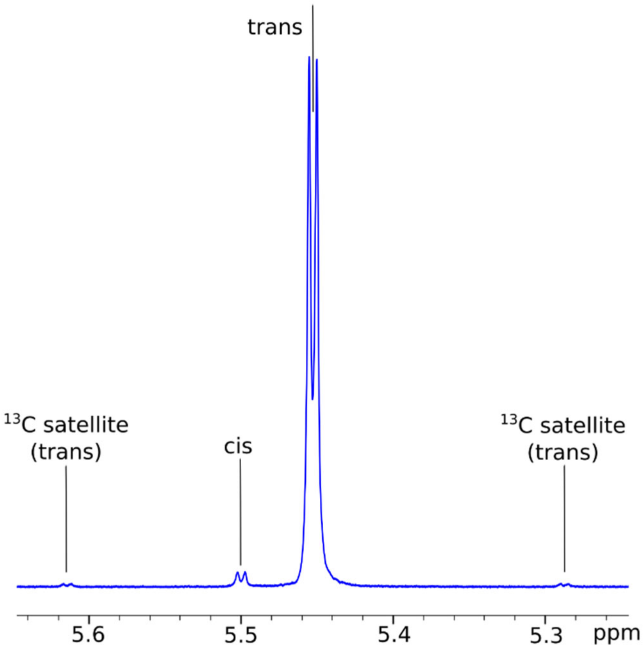

Figure S4. γ-H signals in ^{1}H NMR spectrum of **2**.
